# Database Oriented Big Data Analysis Engine Based on Deep Learning

**DOI:** 10.1155/2022/4500684

**Published:** 2022-08-31

**Authors:** Xiaoran Shang

**Affiliations:** Software School of Liaoning Technical University, Huludao, Liaoning 125105, China

## Abstract

In recent years, with the development of enterprises to the Internet, the demand for cloud database is also growing, especially how to capture data quickly and efficiently through the database. In order to improve the data structure at all levels in the process of database analysis engine, this paper realizes the accurate construction and rapid analysis of cloud database based on big data analysis engine technology and deep learning wolf pack greedy algorithm. Through the deep learning strategy, a big data analysis engine system based on the deep learning model is constructed. The functions of deep learning technology, wolf greedy algorithm, and data analysis strategy in the cloud database analysis engine system are analyzed, as well as the functions of the whole analysis engine system. Finally, the accuracy and response speed of the cloud database analysis engine system are tested according to the known clustering data. The results show that compared with the traditional data analysis engine system with character search as the core, the database oriented big data analysis engine system based on a deep learning model and wolf swarm greedy algorithm has faster response speed and intelligence. The research application is that the proposed engine system can significantly improve the effect of the analysis engine and greatly improve the retrieval accuracy and analysis efficiency of fixed-point data in the database.

## 1. Introduction

With the development of cloud computing, the whole IT basic technology has undergone Earth shaking changes. The deployment of IT facilities has changed from fragmented in the past to centralized and large-scale today. Today, based on cloud computing services, enterprise IT facilities are centralized and large-scale, and the requirements for efficiency and performance are improved. In the past, the data form or application scenario was relatively single. Taking traditional databases as an example, the scenario was mainly concentrated in traditional industries such as finance, operators, government affairs, and so on. With the development of the Internet, mobile Internet, and industrial Internet, all industries are gradually accelerating their development trend of electronization and informatization. The forms of application services are developing in a diversified manner, which makes the data forms and application scenarios of the current industry more and more diversified and puts forward more requirements and challenges to the underlying database capabilities. Cloud computing is the foundation of the rise of a cloud database, which is the database deployed and virtualized in the cloud computing environment. A new method of shared infrastructure developed in the context of cloud computing, which greatly enhances the storage capacity of the database, eliminates the repeated configuration of personnel, hardware, and software, and makes it easier to upgrade software and hardware. Cloud database has the characteristics of high scalability, high availability, multitenancy, and effective distribution of resources. A Cloud database is an ideal choice for personalized data storage needs. At present, enterprise business is increasingly inseparable from the network, so most enterprises have built a relatively perfect cloud private database [1]. However, most of these cloud databases are based on conventional string search engines. Therefore, when enterprises carry out directional data retrieval, their retrieval efficiency is very low [2]. Although the proportion of analog part in digital simulation technology is low, the error rate of language data bytes from analog part is very high [3]. Therefore, while establishing the cloud database, the users of the cloud database also put forward higher requirements for the response speed and retrieval accuracy of the data search engine [4]. In recent years, the rapid development of deep learning technology and wolf pack greedy algorithm can cluster and reorganize according to the characteristics between data after self-learning a large number of data, so it can significantly improve the response speed of its search engine [5]. Based on this, this paper proposes a database oriented big data analysis engine system based on deep learning.

This paper studies the relevant methods of optimizing the cloud database analysis engine and puts forward the cloud database analysis engine method based on big data analysis strategy, deep learning technology, and wolf swarm greedy algorithm, which is mainly divided into four chapters.  Section 1 summarizes the research background and research content framework; Section 2 introduces the construction method of the analysis engine, the current situation, and shortcomings of deep learning application of cloud database and summarizes the pain points of cloud database in the process of establishing analysis engine.  Section 3 introduces the role and overall architecture of big data technology and deep learning network in building database big data analysis engine system.  Section 4 uses the established database analysis engine model to practice a large number of sample data through in-depth learning technology and designs confirmatory experiments to analyze the accuracy and efficiency of the database analysis engine.

The innovation of this paper is that the deep learning strategy and wolf swarm greedy algorithm are used in the process of establishing the database analysis engine. Wolf pack greedy algorithm based on a deep learning strategy can deeply analyze and optimize the hierarchical structure of the database. Solve the cost and efficiency problems of traditional cloud database when using an analysis engine. And, greatly improve the data structure at all levels in the process of database analysis engine and then improve the retrieval efficiency and accuracy involved in the database analysis engine. Experiments show that the data analysis engine system based on the wolf swarm greedy algorithm can significantly improve the effect of the analysis engine. Compared with the traditional big data engine system based on feature analysis, it greatly improves the retrieval accuracy and analysis efficiency of fixed-point data in the database.

## 2. Related Work

The current database big data analysis engine system is mainly based on a character search strategy. Although this engine strategy can quickly improve its internal relevance and accuracy, it is difficult to realize rapid and accurate cloud matching [6]. Han et al. proposed an automatic retrieval data analysis engine model based on a chaotic deep learning algorithm. This engine can realize hierarchical fast retrieval by constructing the underlying logic of different types of data [7]. Javan and Akbari found that the establishment of most database analysis engines still follows the traditional establishment ideas and ignores the internal logical characteristics and differences of data structures. Therefore, the retrieval efficiency of the Enterprise Cloud database can not reach the ideal state [8]. Fraser et al. analyze and adjust the logical path of different database analysis engine systems to make the database reach a state of high accuracy in the process of value analysis of data groups. Experiments show that the database analysis engine method established by this method can well improve the error correction and correction ability of the database in the analysis process [9]. According to the traditional establishment mode and practical analysis experience of cloud big data analysis engine, Romanowski found that the current database has the problem of low matching degree in the process of data analysis engine. Therefore, they developed a new retrieval path optimization algorithm based on big data technology, which can effectively improve its engine search efficiency [10]. According to the multifactor relationship theory in literature, Singh et al. scholars proposed a new database analysis engine method based on multirelationship coupling algorithm, analyzed the relationship degree of different modules in the traditional database structure, and established a multifactor coupling analysis model [11]. Chen et al. have proved through experiments that the retrieval method of big data classification can play a good role in differential retrieval in the database and effectively improve the accuracy of fast retrieval [12]. Karam et al. comprehensively evaluated the databases of e-commerce enterprises from the aspects of the selection of cloud database structure, content classification, and database retrieval ability and simulated the retrieval process by using language data under different language systems [13]. The research results of Deblais et al. show that the analysis engine ability of cloud database based on deep learning algorithm is higher than that of classical cloud database in terms of heterogeneous information [14]. In order to improve the revision of data analysis strategies in the database, Zhang et al. proposed a multidimensional analysis engine retrieval method based on deep learning theory through various research and analysis of different language systems [15]. Wagner and Mccomb scholars have conducted many experiments on the deep learning strategy and found that the deep learning strategy can effectively improve the data analysis speed and engine efficiency in the database [16]. Lyu et al. proposed a new method to establish a multifactor based clustered cloud database analysis engine and realized the optimal determination of data analysis schemes of different dimension types in the database by using the circular analysis of multi-dimensional space [17]. Gui and Gui have proved through practice for many times that the deep learning algorithm can effectively improve the matching degree of retrieval results of cloud database, but there is still the problem of low efficiency of big data analysis, which is greatly affected by network fluctuations [18].

To sum up, most of the analysis systems constructed by the big data analysis engine strategy based on the character search method do not involve the application of intelligent algorithms based on the difference of data logical features [19–21]. On the other hand, although there are many theoretical analysis and Research on the analysis engine of cloud database, there is room for progress in practical application [22, 23]. Therefore, it is of great significance to carry out the research on database oriented big data analysis engine based on deep learning.

## 3. Methodology

### 3.1. Application of Wolf Swarm Greedy Algorithm Based on Deep Learning in Cloud Database Analysis Engine System

**Figure 1:**
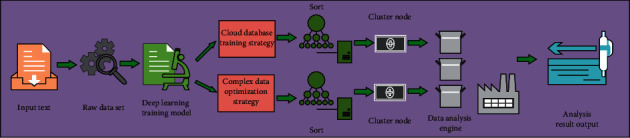
Data analysis principle of wolf pack greedy algorithm based on deep learning.

Deep learning is a complex machine learning algorithm. Its ultimate goal is to enable machines to have the ability of analysis and learning like people, and to recognize text, image, sound, and other data [24]. Its principle is shown in Figure 1. Under the background of the Internet era, there are a large number of complex data optimization problems in the design and process of a cloud database, and these problems are difficult to solve. Therefore, using new optimization methods such as deep learning and deep learning to solve these problems has become people's research goal [25, 26]. There is a common algorithm in deep learning, the greedy algorithm. One of the typical representatives of this kind of algorithm is the wolf swarm greedy algorithm. Although the traditional wolf clustering method enhances the adaptability of the algorithm, the algorithm only clusters according to the fitness. The distribution of wolves in the solution space is not considered. In order to make the division of subgroups reasonable, in addition to selecting the head wolf of the subgroup according to the size of fitness, it is also necessary to screen according to the distance between the head wolves, so as to maximize the distance between subgroups after clustering. In addition, it is also necessary to dynamically adjust the subgroup division according to different environments and changes in the distribution of wolves, so as to improve the dynamic adaptability of the algorithm. Based on this, this paper uses the wolf swarm greedy algorithm to optimize the database in data analysis and data engine. In the process of constructing a cloud database, the algorithm mainly realizes the high-efficiency coincidence analysis of data through the determination of database structure and the prediction of function results.

### 3.2. Data Analysis Process of Cloud Database Big Data Analysis Engine System

**Figure 2:**
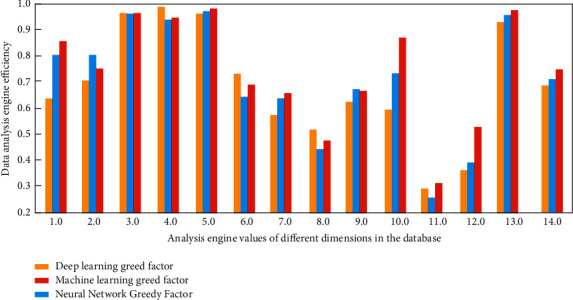
Simulation analysis results of wolves greedy optimization algorithm on cloud database output prediction.

In the process of cloud database analysis and engine search, the wolf swarm greedy algorithm based on deep learning uses multiple operations such as selection, crossover, and mutation to explore the optimal fitness value l levels. In the process of database analysis, first, the initial weight and critical value of the network should be adjusted through the optimal fitness value obtained by deep learning and the wolf pack greedy algorithm. Greedy search is used to select the optimal test step by step. The effectiveness of the algorithm is verified by the optimization selection cases of binary and multivalued tests. The time complexity of the algorithm is analyzed theoretically. Theoretical and case analysis results show that the algorithm can effectively mine the fault detection and isolation capabilities of the test. It is also applicable to the optimal selection of binary and multivalued tests isolated to replacement units and specific fault modes. After training, the output value of the function should be analyzed and compared with the engine, so as to form a deep learning model with structure, weight and threshold determined, and then use the data sample set to train the database; the output of the cloud database is predicted, and the predicted simulation analysis results are shown in Figure 2.

As can be seen from Figure 2, with the increase of analysis engine values in different dimensions in the database, the prediction results of the wolf pack greedy algorithm under three different greedy factor mechanisms on simulation data are significantly different, showing the law of fluctuation of accurate coincidence rate with the increase of calculation times, And, the wolf swarm greedy algorithm based on deep learning greedy factor has the highest efficiency and comprehensive degree.

After completing the above steps, it is necessary to take the absolute value of the error between the predicted output and the expected output as the individual fitness value *W* and the weight value *f*, and the calculation formula is

(1) $$\begin{array}{c} \begin{array}{c} W=\frac{\sqrt{s}\overset{n}{\underset{i}{\sum }}aby^{k}}{n^{s}+n^{s-a}+n^{s-b}+1}\text{,}\\ f=\frac{\sqrt{H_{i}^{2}-y_{i}^{2}/ik}}{Wn^{s}+Wn^{s-a}+n^{s-b}+W}. \end{array} \end{array}$$

**Figure 3:**
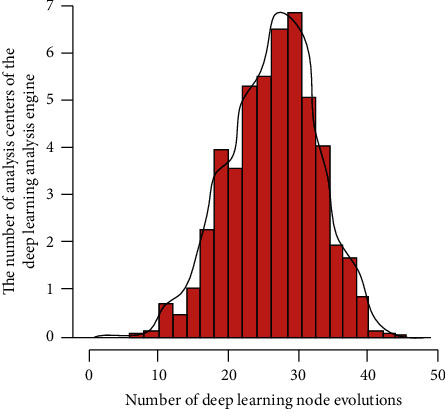
The relationship between the number of neuron node evolution of deep learning neural network algorithm and the learning center.

Here, *n* is the number of nodes in the output network of the hidden layer and the output layer, and *y*_*i*_ is the predicted output of the *i* node of the output network; *H*_*i*_ is the measured output of the *i* node; *k* is the coefficient. Let the engine factor function *p*_*i*_ of each data segment *i* be:

(2) $$\begin{array}{c} \begin{array}{c} T_{i}=\frac{\sqrt{dF_{i}/dk\overset{n}{\underset{i=1}{\sum }}F_{i}^{2}}}{H_{i}^{2}}\text{,}\\ p_{i}=\int \frac{f_{i}-N}{\sqrt{kf}}, \end{array} \end{array}$$

where *T*_*i*_ is the engine analysis value of the cloud data segment and *k* is the coefficient; *N* is the maximum value for engine analysis. The engine analysis method of *k* data body *p*_*kj*_ and *l* data body *p*_*ij*_ in *j* bit is as follows:

(3) $$\begin{array}{c} \begin{array}{c} p_{kj}=\overset{n}{\underset{i}{\sum }}bp_{kj}+p_{lj}\frac{1}{n}{\bar{p}}_{kj-1}+{\bar{p}}_{lj-1}\text{,}\\ p_{ij}=\frac{\sqrt{\overset{n}{\underset{i}{\sum }}\sqrt{p_{ij}+p_{kj}b}/1-b}}{1+b}, \end{array} \end{array}$$

where *b* is a random number between [0, 1]. In terms of multidimensional analysis of cloud database, the discrimination methods for the *i* database analysis engine are as follows:

(4) $$\begin{array}{c} p_{ij}=\frac{\sqrt{W\left(g\right)/p_{ij}+\text{(}p_{ij}-{p2}_{\max}}}{W\left(g\right)*p_{ij}}\text{,} \end{array}$$

where *p*_max_ is the upper bound of data *p*_*ij*_; *p*_min_ is the lower bound of data *p*_*ij*_;

(5) $$\begin{array}{c} W\left(g\right)=\frac{\sqrt{r_{2}}r_{2}{\left(p_{\max}+p_{\max}\right)}^{3}}{p_{\max}-p_{\max}}\text{,} \end{array}$$

where *r*_2_ is a random number between [0, 1]. In this process, the simulation analysis results of the big data analysis engine under the deep learning strategy are shown in Figure 3.

It can be seen from Figure 3 that with the increase of the evolution times of deep learning nodes, the deep learning centers in the wolf swarm greedy algorithm based on deep learning also gradually increase, and under the same evolution times, the threshold setting deep learning wolf swarm greedy algorithm method has the most learning centers, because the deep learning wolf swarm greedy algorithm is used to optimize the cloud database, It can effectively combine the advantages of big data and depth algorithms, and overcome their defects to a certain extent. Introducing this algorithm into cloud database feature data diagnosis can optimize the cloud database feature data diagnosis method. The greedy algorithm searches in sequence in multiple task stages and seeks the local optimal solution in the current situation in each stage. Make successive greedy choices in an iterative manner, and each time you make a choice, the problem to be solved will be transformed into a smaller scale problem, and finally the overall optimal solution or approximate solution will be obtained. The algorithm mostly puts forward test evaluation criteria based on the amount of information, and it is difficult to obtain the information of equipment failure rate in practical application. Even if the acquired failure rate data are mostly empirical data, the accuracy is still questionable. The algorithm is mostly applicable to the optimization selection of binary rather than multivalued testing, and it is impossible to perform the test optimization calculation isolated to Ru.

### 3.3. Simulation Process of Discrimination Analysis of Wolf Swarm Greedy Algorithm Based on Deep Learning in Cloud Database

**Figure 4:**
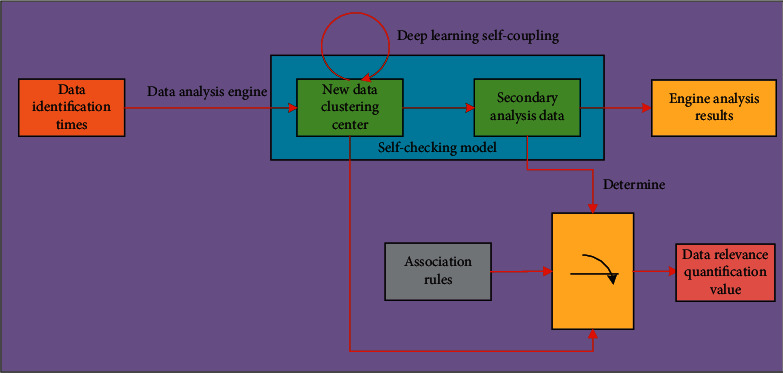
The process of analyzing the number of data analysis groups in the cloud database.

In the process of identifying and analyzing the data in the cloud database, firstly, the starting point of the database needs to be found through the wolf pack greedy algorithm, and the wolf pack greedy algorithm method is used for correlation analysis. In the data groups of different dimensions, with the change of data identification times, the process of analyzing the number of data analysis groups in the cloud database is shown in Figure 4.

**Figure 5:**
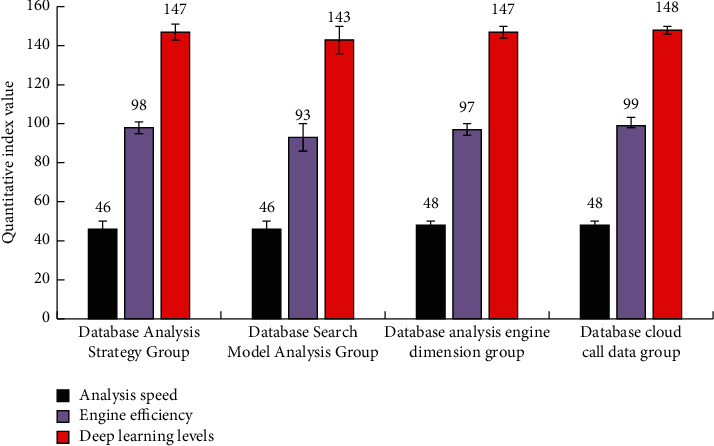
Simulation analysis and statistical results of data analysis and information extraction by cloud database.

In the process of analyzing the identified data, the key point of the analysis method is to compare the dimensions of different types of data. Multidimensional data analysis is based on a database or data warehouse, and its final data comes from the underlying database system. Multidimensional data analysis is more suitable for data analysis and processing based on a data warehouse. Here, combined with the important efficiency evaluation index OEE in the production field, we study how to use multidimensional ideas and methods to analyze. After analyzing the data of the same dimension, its internal relevance is very obvious and can be calculated according to requirements; if the data dimensions are different, the low latitude data can be expanded and supplemented to make the dimensions of the two groups of data the same, and then the data operation can be carried out. The simulation analysis and statistical results of data analysis and information extraction are shown in Figure 5.

As can be seen from Figure 5, because there are many messy feature data, in the process of analyzing the engine, the similarity of the quantitative evaluation indicators of the internal relevance of the data of the four groups is poor. This is because the cloud database needs a lot of data in the process of “deep learning,” so its similarity is not high, but the overall change trend is still very close. In order to reduce the analysis and engine search time of the cloud database, the system reduces the dimension of the fixed-point data extracted by the above analysis through discrete cosine transform.

If the fixed sequence of the analysis engine data group in the cloud database is a group of characteristic data of *Y*(*n*) and *n*=0,1,…, *N* − 1, the dimension reduction formula is as follows:

(6) $$\begin{array}{c} \begin{array}{c} Y_{c}\left(0\right)=\frac{Y\left(0\right)/N\overset{N-1}{\underset{n=0}{\sum }}8\;\sin^{-1}\;Y\left(n\right)}{1-\cos^{3}\;Y\left(n\right)}\text{,}\\ Y_{c}\left(k+1\right)=\frac{\left(1-\cos^{3}\;Y\left(n\right)\right)\sqrt{k/N}\overset{N-1}{\underset{n=0}{\sum }}e^{\left(2n+1\right)k\pi /2N\sqrt{x\left(n\right)}}}{1-\sin^{3}\;Y\left(n\right)},k=1,2,\ldots N-1\text{.} \end{array} \end{array}$$

Next, the characteristic data in the cloud database can be further determined according to the number of hidden nodes *x*(*n*) and hierarchy points:

(7) $$\begin{array}{c} \begin{array}{c} Y\left(n\right)=\frac{\sqrt{\sum Y_{c}\left(k\right)\sin\left(2n+1\right)k\pi /2N}}{1-\cos^{3}\;Y\left(n\right)}\text{,}\\ Y\left(n\right)=\frac{\sqrt{\overset{n}{\underset{i=1}{\sum }}Y_{c}\left(k\right)*Y_{c}\left(0\right)/i^{2}}}{i^{2}+2}, \end{array} \end{array}$$

where *n*represents the number of nodes of the wolf swarm greedy algorithm; *i* represents the number of bits of the wolf swarm greedy algorithm; *k* represents a nonzero constant.

In the process of data analysis and engine extraction, the learning process of cloud database converges slowly. To solve this problem, based on the wolf pack greedy algorithm, the additional momentum algorithm is adopted, and its formula at different times is

(8) $$\begin{array}{c} \begin{array}{c} R\left(k\right)=\frac{\Delta R{\left(k\right)}^{2}+\int a\left[dR\left(2k-1\right)-dR\left(2k+1\right)\right]}{\sqrt{1+\int a\left[dR\left(2k-1\right)-dR\left(2k+1\right)\right]}}\text{,}\\ R\left(k+1\right)=\frac{\sqrt{\Delta R{\left(k{\text{)}}^{2}-\Delta R\text{(}2k-1\right)}^{2}/k}}{1+k}\text{,}\\ R\left(2k+1\right)=\frac{\sqrt{\Delta R{\left(2k+1{\text{)}}^{2}-\Delta R\text{(}2k+1\right)}^{2}/2k}}{1+2k}, \end{array} \end{array}$$

where Δ*R*(*k*), *R*(2*k* − 1) and *R*(2*k*+1) are the data group numbers corresponding to *k*, 2*k* − 1 and 2*k*+1 respectively; *a* is the efficiency constant of the analysis engine.

## 4. Result Analysis and Discussion

### 4.1. Simulation Experiment and Data Analysis

**Figure 6:**
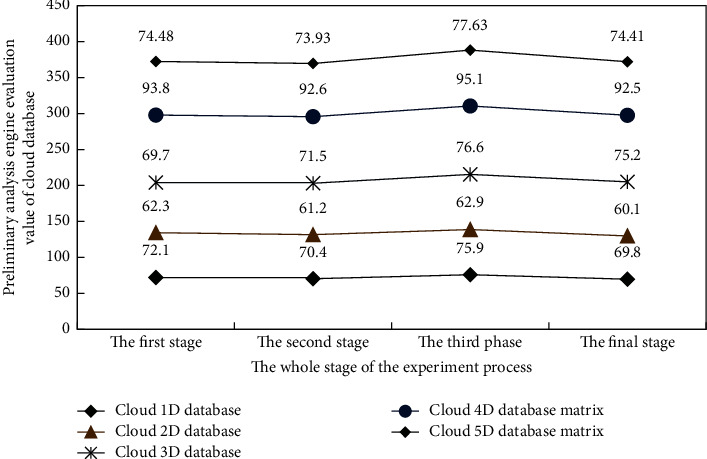
Experimental preliminary analysis results of the efficiency of analysis engines for different types of cloud databases.

In the specific experimental process, Matlab 2020b programming software is used to start the experimental process. Before the experiment, various parameters in the algorithm need to be set in advance. For convenience, this study sets the initial value of the greedy iterative algorithm of the deep learning algorithm as 10, the crossover probability as 50%, and the mutation probability of the artificial deep learning algorithm as 10%. The maximum algebraic value is 50. Through greedy iterative depth learning and quantitative characterization depth learning, a large number of sample data are trained, multiple experimental analysis processes are carried out, and relevant experimental data are recorded. The preliminary experimental analysis results are shown in Figure 6.

As can be seen from Figure 6, under the deep learning algorithm, the Wolf swarm greedy algorithm is used for different types of cloud databases (the initial greedy factor set by the algorithm is the same), and its reliability to the results of five groups of experimental data is different because the data collection samples referred to in the experimental process include deep learning training samples and simulation experiment test samples. The training samples of deep learning are known, and the deep learning algorithm will be in the “guidance” of category training samples The test samples are tested, judged, and classified and grounded in different branch points. The response of the deep learning algorithm in the location of grounded feature data in the cloud database is judged by artificially manufacturing feature data nodes. Therefore, the analysis strategies and search engine rules of the corresponding cloud database are also different, This also shows that the database oriented big data analysis engine system based on deep learning can effectively analyze and retrieve different data in the cloud database.

### 4.2. Result Analysis

**Figure 7:**
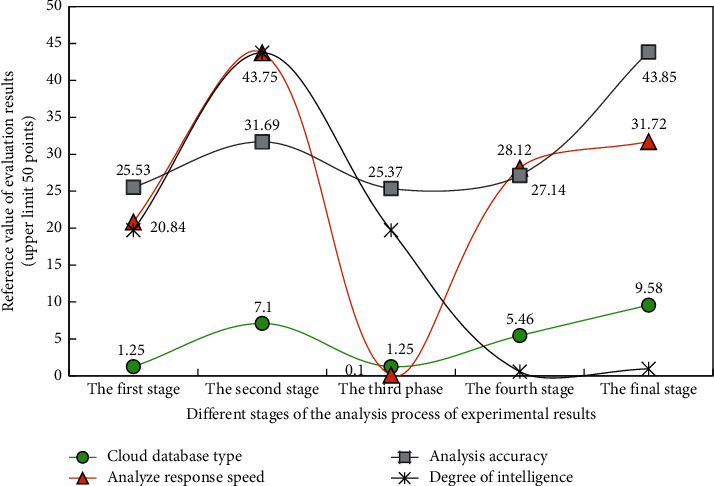
The result of the final comprehensive analysis of the analysis engine on the experimental data.

According to the preliminary experimental data, in the case of each cluster number, with the increasing number of cluster classes, the advantages of the wolf swarm greedy algorithm based on deep learning in the analysis engine of the cloud database are gradually displayed. In order to further analyze the factors affecting the analysis engine, the final error degree of the analysis engine is analyzed for the experimental results, The analysis results are shown in Figure 7.

It can be seen from the analysis results in Figure 7 that when the wolf swarm greedy optimization algorithm based on deep learning solves the target problems in the cloud database during the experiment, the final data analysis accuracy remains at a high level, and when the analysis times are higher than the fourth stage, it performs well in processing the characteristic data in the database in the branch point. The speed and accuracy of the search engine for the specified data have been significantly improved, so the accurate matching of the characteristic data combined with the data group can be realized. Moreover, with the improvement of the analysis stage, the wolf swarm greedy algorithm based on deep learning shows a law that is very consistent with the experimental expectation in the analysis of cloud database.

## 5. Conclusion

With the development of the Internet era, cloud database is becoming more and more important to the development of enterprises, especially in the data analysis of database and search engine. Based on this, this paper establishes a multi-level cloud database data analysis engine system based on big data and deep learning. Firstly, it introduces the development status of different types of cloud databases and the pain points of research progress. Combined with the shortcomings of existing research, a cloud database big data analysis engine system based on a wolf swarm greedy algorithm based on deep learning is proposed. Secondly, it introduces the role of deep learning technology, wolf greedy algorithm, and data analysis strategy in the cloud database analysis engine system, as well as the functionality of the overall analysis engine system. Finally, the multidimensional diagnosis simulation analysis of the cloud database is carried out. The experiment shows that the data analysis engine system established by the Wolf swarm greedy algorithm based on deep learning can significantly improve the effect of the analysis engine and greatly increase the retrieval accuracy and analysis efficiency of fixed-point data in the database. However, this paper only focuses on the core modules of the cloud database analysis engine system in the construction process and does not take the differentiation factors of specific use scenarios into account. Therefore, the cloud database data analysis engine system established by this method needs to be further studied. In addition, exploring wolves is to randomly select *h* directions and then explore. There is no communication between them, and there will be repetition in the exploration space. At present, it is difficult for the wolf swarm intelligence algorithm to find the global optimal solution. Constantly exploring swarm intelligence optimization algorithm has become a hot topic for many scholars.

## Data Availability

The data used to support the findings of this study are available from the author upon request.

## References

[1] Tang L., Zhang C., Li L., Wang S. (2020). A multi-scale method for forecasting oil price with multi-factor search engine data. *Applied Energy*.

[2] Uhrin M., Huber S. P., Yu J., Marzari N., Pizzi G. (2021). Workflows in AiiDA: engineering a high-throughput, event-based engine for robust and modular computational workflows. *Computational Materials Science*.

[3] Hong S., Jeong D., Hwang H. S. (2020). Constraining cosmology with big data statistics of cosmological graphs. *Monthly Notices of the Royal Astronomical Society*.

[4] Cabrera V. E., Barrientos-Blanco J. A., Delgado H., Fadul-Pacheco L. (2020). Symposium review: real-time continuous decision making using big data on dairy farms. *Journal of Dairy Science*.

[5] Yang J., Wen J., Jiang B., Wang H. (2020). Blockchain-based sharing and tamper-proof framework of big data networking. *IEEE Network*.

[6] Talley K. R., Bauers S. R., Melamed C. L. (2019). COMBIgor: data-analysis package for combinatorial materials science. *ACS Combinatorial Science*.

[7] Han R., Liu C. H., Li S. (2020). Accelerating deep learning systems via critical set identification and model compression. *IEEE Transactions on Computers*.

[8] Javan M. S., Akbari M. K. (2019). SmartData 4.0: a formal description framework for big data. *Journal of Supercomputing*.

[9] Fraser K. C., Lundholm Fors K., Eckerström M., Ohman F., Kokkinakis D. (2019). Predicting MCI status from multimodal language data using cascaded classifiers. *Frontiers in Aging Neuroscience*.

[10] Romanowski A. (2019). Big data-driven contextual processing methods for electrical capacitance tomography. *IEEE Transactions on Industrial Informatics*.

[11] Singh A., Garg S., Kaur K., Batra S., Kumar N., Choo K. K. R. (2019). Fuzzy-folded bloom filter-as-a-service for big data storage in the cloud. *IEEE Transactions on Industrial Informatics*.

[12] Chen Y. T., Sun E. W., Lin Y. B. (2019). Coherent quality management for big data systems: a dynamic approach for stochastic time consistency. *Annals of Operations Research*.

[13] Karam C. S., Coie L. A., Javitch J. A. (2020). Small flies meet big data: genetic convergence of neurodevelopmental disorders modeled in*Drosophila*. *American Journal of Psychiatry*.

[14] Deblais L., Kathayat D., Helmy Y. A., Closs G., Rajashekara G. (2020). Translating ‘big data’: better understanding of host-pathogen interactions to control bacterial foodborne pathogens in poultry. *Animal Health Research Reviews*.

[15] Zhang L., Zhang H., Jiang Y. (2020). Intelligent and reliable deep learning LSTM neural networks-based OFDM-DCSK demodulation design. *IEEE Transactions on Vehicular Technology*.

[16] Wagner K. H., Mccomb S. M. (2020). Optical rectifying linear units for back-propagation learning in a deep holographic convolutional neural network. *IEEE Journal of Selected Topics in Quantum Electronics*.

[17] Lyu B., Hu Y., Zhang W. (2019). Fusion method combining ground-level observations with chemical transport model predictions using an ensemble deep learning framework: application in China to estimate spatiotemporally-resolved PM_2.5_ exposure fields in 2014–2017. *Environmental Science & Technology*.

[18] Gui S., Gui R. (2019). Utilizing wavelet deep learning network to classify different states of task-fMRI for verifying activation regions. *International Journal of Neuroscience*.

[19] Tong X., Brandt M., Hiernaux P. (2020). The forgotten land use class: mapping of fallow fields across the Sahel using Sentinel-2. *Remote Sensing of Environment*.

[20] Liu S., Gao Z., Zhang J. (2020). Deep denoising neural network assisted compressive channel estimation for mmWave intelligent reflecting surfaces. *IEEE Transactions on Vehicular Technology*.

[21] Satti F. A., Ali T., Hussain J., Khan W. A., Khattak A. M., Lee S. (2020). Ubiquitous Health Profile (UHPr): a big data curation platform for supporting health data interoperability. *Computing*.

[22] Irmak E. (2021). Multi-classification of brain tumor MRI images using deep convolutional neural network with fully optimized framework. *Iranian Journal of Science and Technology - Transactions of Electrical Engineering*.

[23] Mkkn A., Yl B., Fl D. (2019). Pathological image compression for big data image analysis: application to hotspot detection in breast cancer - ScienceDirect. *Artificial Intelligence in Medicine*.

[24] Lin Y., Tu Y., Dou Z. (2020). An improved neural network pruning technology for automatic modulation classification in edge devices. *IEEE Transactions on Vehicular Technology*.

[25] Shaw H. J., Lin C. K. (2021). Marine big data analysis of ships for the energy efficiency changes of the hull and maintenance evaluation based on the ISO 19030 standard. *Ocean Engineering*.

[26] Liu F. (2022). Language database construction method based on big data and deep learning. *Alexandria Engineering Journal*.

